# Evaluation of non-response to the In-Center Hemodialysis Consumer Assessment of Healthcare Providers and Systems (ICH CAHPS) survey

**DOI:** 10.1186/s12913-018-3618-4

**Published:** 2018-10-19

**Authors:** Taimur Dad, Hocine Tighiouart, Joshua J. Fenton, Eduardo Lacson, Klemens B. Meyer, Dana C. Miskulin, Daniel E. Weiner, Michelle M. Richardson

**Affiliations:** 1 Tufts Medical Center and Tufts University School of Medicine, 800 Washington Street, Box 391, Boston, MA 02111 USA; 2 Institute for Clinical Research and Health Policy Studies, Tufts Medical Center, Boston, MA USA; 3 Biostatistics, Epidemiology and Research Design (BERD) Center, Tufts Clinical and Translational Science Institute, Tufts University, Boston, MA USA; 4 Department of Family and Community Medicine, Center for Healthcare Policy and Research, University of California, Davis, Sacramento, CA USA; 5 Dialysis Clinic Incorporated, Nashville, TN USA

**Keywords:** Hemodialysis, CAHPS, ICH CAHPS, Patient reported outcome, Patient satisfaction, Patient experience, Survey

## Abstract

**Background:**

The In-Center Hemodialysis Consumer Assessment of Healthcare Providers and Systems (ICH CAHPS) Survey is the first patient reported outcome measure included in the U.S. Medicare End Stage Renal Disease Quality Incentive Program. Administered twice yearly, it assesses in-center dialysis experience and survey responses are tied to dialysis facility payments. Low response rates, currently approximately 35%, raise concern for possible underrepresentation of patient groups.

**Methods:**

Cross-sectional analysis of survey administration in 2012 to all in-center hemodialysis patients in Dialysis Clinic, Inc. (DCI) facilities nationally over 18 years old who received hemodialysis at their facility for at least 3 months. Patient-level covariates included demographic, clinical, laboratory, and functional characteristics. Random effects multivariable logistic regression was used to assess survey non-response.

**Results:**

Among 11,055 eligible patients 6541 (59%) were non-responders. Of the remaining 4514 responders, 549 (14%) surveys were not usable due to presence of proxy help or incomplete responses. Non-responders were more likely to be men, non-white, younger, single, dual Medicare/Medicaid eligible, less educated, non-English speaking, and not active on the transplant list; non-responders had longer ESRD vintage, lower body mass index, lower serum albumin, worse functional status, and more hospitalizations, missed treatments, and shortened treatments. Similar associations were found using more parsimonious multivariable analyses and after imputing missing data.

**Conclusions:**

Non-responders to the ICH CAHPS significantly differed from responders, broadly spanning individuals with fewer socioeconomic advantages and greater illness burden, raising limitations in interpreting facility survey results. Future research should assess reasons for non-response to improve ICH CAHPS generalizability and utility.

**Electronic supplementary material:**

The online version of this article (10.1186/s12913-018-3618-4) contains supplementary material, which is available to authorized users.

## Background

Patient experience is an integral part of patient-centered care. Multiple factors influence patient experience, including characteristics of the facility, interactions with care teams, patient expectations, and response to or complications of treatment. Interest in measuring patient experience dates to Health Effectiveness Data and Information Set (HEDIS) measures evaluating this in the early 1990s in the United States [1]. However, non-response bias and low response rates complicate measurement of patient experience [2–4].

The US Agency for Healthcare Research and Quality (AHRQ) developed consumer assessment surveys starting in the 1990s. The Centers for Medicare and Medicaid Services (CMS) in conjunction with AHRQ began developing the In-Center Hemodialysis Consumer Assessment of Healthcare Providers and Systems (ICH CAHPS) survey in 2004 [5]. After field testing in 2005, ICH CAHPS was endorsed by the National Quality Forum (NQF) in 2007 and was incorporated into the End Stage Renal Disease Quality Incentive Program (ESRD QIP) as the first patient reported outcome measure in 2014 [6–8]. Mandatory twice yearly survey administration began in 2016, and facilities with at least 30 annual responses are subject to financial penalties for lower patient experience scores.

Critically, there may be informative differences among patients who complete and do not complete the ICH CAHPS survey that may result in misrepresentation of overall patient experience at a dialysis facility; however, despite its incorporation into value-based payments several years ago, little is known about characteristics of responders and non-responders. Response rates during development and validation of ICH CAHPS were only 46% [8], despite conditions being optimized during this development process. Response rates have continued to drop since the ICH CAHPS has been implemented in the clinical setting, even while the financial and public reporting importance of this assessment of patient experience has increased [9, 10]. As in other areas of medicine, understanding presence of bias and the subsequent generalizability of a test is of utmost importance when interpreting test results and prior to implementing change. Accordingly, we performed the first step in the evaluation of non-response bias by exploring patient characteristics associated with non-response to the ICH CAHPS survey administered in 2012 to patients treated at Dialysis Clinic, Inc. (DCI) facilities nationally.

## Methods

### Study population

Per 2012 AHRQ guidelines, ICH CAHPS eligible patients consisted of all in-center HD patients at least 18 years old who had been at their facility for at least 3 months. Responses from eligible patients were deemed usable only if patients indicated receiving no proxy help and at least 50% of pre-defined key questions were answered (Additional file 1: Box 1) [11].

### Survey

The ICH CAHPS survey administered in 2012 had 58 questions and was available in English and Spanish. Responses were grouped into three composite scores and three global rating scales. The three composite scores were ‘*Nephrologists’ Communication and Caring*’, ‘*Quality of Dialysis Center Care and Operations*’, and ‘*Providing Information to Patients*’; these composite scores were derived from questions that used either ‘*never/sometimes/usually/always*’ responses or ‘*yes/no*’ responses. The three global rating scales rated nephrologists, dialysis center staff, and the dialysis facility on a scale of 0-10 (with 0 being worst and 10 being best). The remaining survey questions asked about demographic characteristics, comorbid medical conditions, and whether or not help was received in answering the survey questions.

### Survey administration

Dialysis facilities were required to select third party vendors to administer the ICH CAHPS survey. DCI’s survey vendor followed AHRQ guidelines for survey administration, data collection, and data submission. Before the survey administration period, DCI in-center HD facilities received staff and patient education materials describing AHRQ survey administration requirements. AHRQ requirements did not allow dialysis provider, facility staff or physician involvement in survey administration or in the collection of results. As instructed by the survey vendor, on August 3, 2012 DCI created a data file of eligible patients from its electronic medical information system containing mailing addresses, telephone numbers, and primary language. Approximately 10 days later, the survey vendor mailed patients a pre-notification letter on DCI letterhead, signed by a member of the DCI executive team. The letter informed patients that they would receive a survey regarding the care they received at their dialysis facility and that their responses were very important. One week later, ICH CAHPS surveys were mailed to all potentially eligible patients by the survey vendor. Patients were instructed to mail completed surveys directly back to the survey vendor in pre-paid and addressed envelopes. Two weeks after the first survey mailing, the survey vendor sent a reminder letter to non-responders to the first mailing, and another copy of the survey 30 days after the first survey mailing. In October, the survey vendor contacted patients who had not replied to either of the mailed surveys by telephone up to three times over a 4-week period.

### Study design

**Fig. 1:**
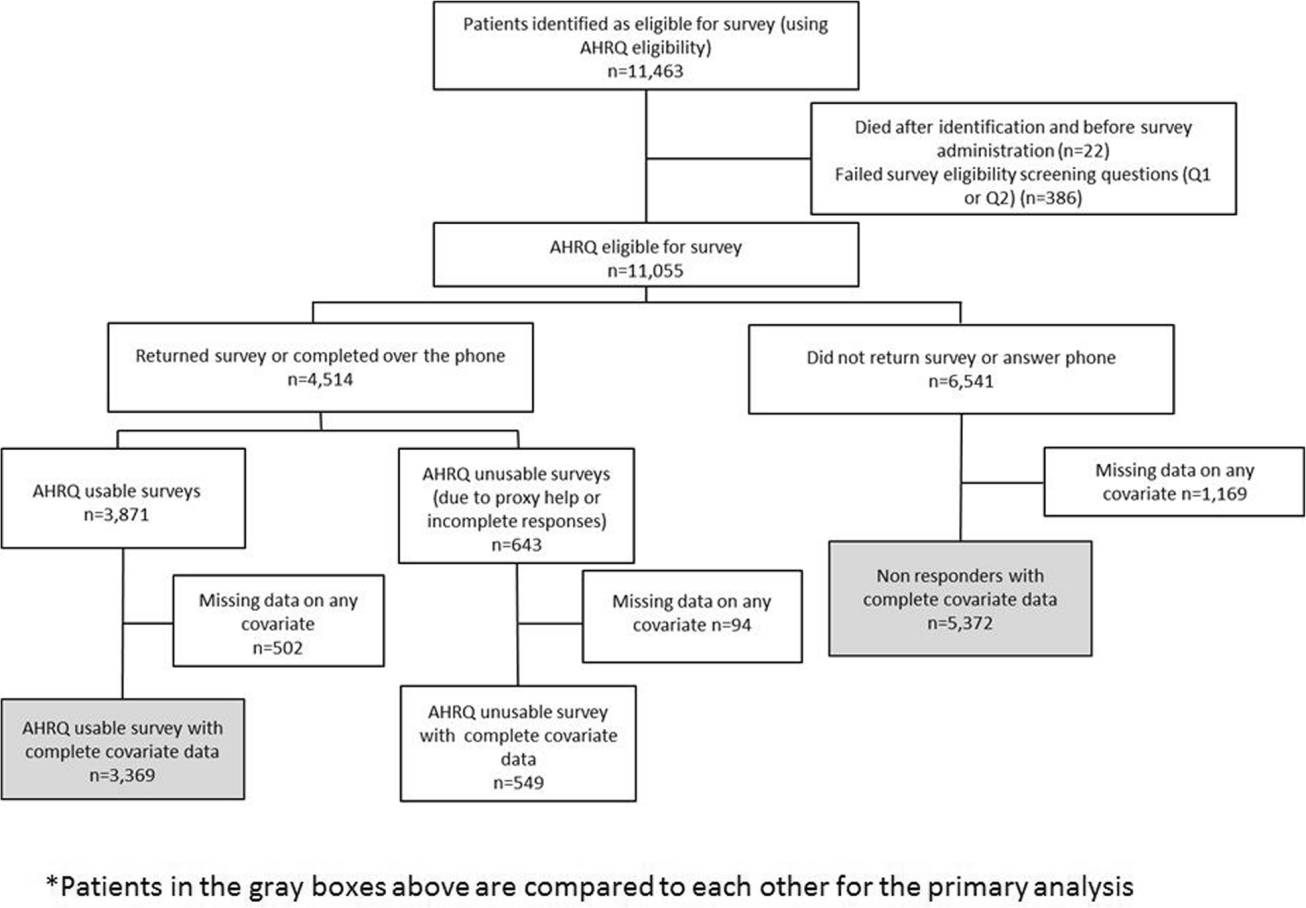
Flow diagram

DCI has over 200 dialysis facilities nationally. Their survey vendor provided patient-level data from the 2012 survey period to DCI exclusively for quality improvement and research purposes under a signed Respondent Identifiable Information Disclosure Agreement. A member of the DCI information technology team who was independent from the research team merged survey data to individual patient DCI electronic medical data and de-identified the dataset. The primary study outcome was non-response to the ICH CAHPS survey. In primary analyses, only surveys meeting AHRQ’s definition of usable (no proxy help and answers to at least 50% of pre-defined key questions) were included (Fig. 1). In secondary analyses, survey response was defined using an “expanded usable” criteria which included AHRQ usable surveys as well as surveys without 50% of pre-defined key questions answered and surveys indicating proxy help (Additional file 1: Figure S1a). For surveys indicating proxy help, we only included surveys where the patient checked off receiving help from a family member or friend and checked off any of the following describing the help they received: “Read the questions to me,” “Wrote down the answers I gave,” or both. This definition is more consistent with current ICH CAHPS scoring rules.

To account for missing data, we performed multiple imputation of missing covariates for use in sensitivity analyses. Following imputation, we compared patients with AHRQ usable responses to non-responders (Additional file 1: Figure S1b), and we compared the “expanded usable” group of responders to non-responders (Additional file 1: Figure S1c). The study was approved by the Tufts Medical Center Investigational Review Board.

### Clinical characteristics

Patient clinical characteristics ascertained from DCI medical records included patient demographics, medical records, clinical variables, information on HD treatments, and functional assessments. Since the precise date of survey completion is not known, all covariate data were taken from August 2012 (when eligible patients were identified by DCI and information was sent to the survey vendor). All laboratory analyses were performed at the central DCI laboratory in Nashville, TN. For missing August laboratory data, we used the last non-missing value within 3 months prior to August 2012. Specifically for missing vascular access data in August, we used the most frequently used vascular access in May, June and July 2012. Unexcused absence was defined as missing an entire HD treatment that was not rescheduled and for which a reason (e.g. hospitalization) was not available; shortened treatments were defined as at least one treatment being shorter than prescribed by 15 min or more, and hospitalizations were defined as any hospital stay. Body mass index (BMI) was calculated using the last estimated dry weight ordered by the patient’s nephrologist. Data on functional covariates including ability to ambulate, ability to transfer, falls in the past month, activities of daily living (ADL) score, and nursing home residence were obtained from the nursing assessment most proximate to the survey administration period. The ADL score was derived from 8 questions from the nursing assessment evaluating the patient’s ability to bathe, dress, feed, use the toilet, shop for groceries, prepare meals, do housework, and take medications; 1 point was given for each activity that the patient could independently perform, and 0 points were given if assistance of any type was needed.

### Statistical analysis

We used a random intercept two-level logistic regression model with patients nested within dialysis facilities to model the probability of non-response using the AHRQ definition. The Intra-class correlation (ICC) was calculated using the latent variable model approach [12]. The unobserved patient variable follows a logistic distribution with individual level variance V_I_ equal to π^2^/3. On this basis, the ICC is calculated as ICC=V_A_/(V_A_ + π^2^/3) where V_A_ is the facility residual variance on the logistic scale. Models were fitted sequentially starting with a parsiminous model using primarily demographic data; subsequent models added clinical and functional data. Secondary analyses used the same covariates in the “expanded usable” cohort defined above. Multivariate multiple imputation for missing covariates was performed using chained equations, averaging five models using Rubin’s rule [13]. The imputation model included response status and variables listed in Table 1, with the exception of ability to ambulate, ability to transfer, history of falls, ADL score, and nursing home residence. To account for the multilevel nature of the data, the imputation model included dummy variables for each dialysis facility. We checked for functional forms of all continuous variables using restricted cubic splines in the rms package in R. There were no statistically significant deviations from linearity for any continuous variable. To measure the overall importance of each variable in the multivariable model, we plotted the ranked Chi-squared minus degrees of freedom for each variable [14]. Analyses were performed using SAS Enterprise Guide (Version 7.12, Cary, NC) and R language (version 3.3.1, R Foundation for Statistical Computing, Vienna Austria).

**Table 1:** Baseline characteristics

	Total (*n* = 9290)	AHRQ usable surveys (*n* = 3369, 36%)	Proxy help/ incomplete responses (*n* = 549, 6%)	AHRQ non- responses (*n* = 5372, 58%)
Age	61.1 ± 14.8	62.1 ± 13.9	68.0 ± 13.1	59.8 ± 15.3
Female Sex	4068 (43.8%)	1547 (45.9%)	215 (39.2%)	2306 (42.9%)
Race				
Black	4126 (44.4%)	1294 (38.4%)	188 (34.2%)	2644 (49.2%)
White	4486 (48.3%)	1917 (56.9%)	340 (61.9%)	2229 (41.5%)
Other	678 (7.3%)	158 (4.7%)	21 (3.8%)	499 (9.3%)
Hispanic	637 (6.9%)	176 (5.2%)	39 (7.1%)	422 (7.9%)
Cause of ESRD				
Diabetes	4015 (43.2%)	1357 (40.3%)	286 (52.1%)	2372 (44.2%)
Hypertension	2630 (28.3%)	960 (28.5%)	149 (27.1%)	1521 (28.3%)
Other	2645 (28.5%)	1052 (31.2%)	114 (20.8%)	1479 (27.5%)
Marital status				
Married	3555 (38.3%)	1465 (43.5%)	295 (53.7%)	1795 (33.4%)
Divorced/Separated	1947 (21.0%)	694 (20.6%)	76 (13.8%)	1177 (21.9%)
Widowed	1476 (15.9%)	476 (14.1%)	104 (18.9%)	896 (16.7%)
Single	2312 (24.9%)	734 (21.8%)	74 (13.5%)	1504 (28.0%)
Education Level				
Grade School	1221 (13.1%)	271 (8.0%)	127 (23.1%)	823 (15.3%)
High School	5679 (61.1%)	2082 (61.8%)	354 (64.5%)	3243 (60.4%)
College/Post Graduate	2390 (25.7%)	1016 (30.2%)	68 (12.4%)	1306 (24.3%)
English speaker	8992 (96.8%)	3326 (98.7%)	528 (96.2%)	5138 (95.6%)
Nursing home resident	682 (7.3%)	92 (2.7%)	40 (7.3%)	550 (10.2%)
Insurance				
Medicare/Medicaid	3303 (35.6%)	959 (28.5%)	179 (32.6%)	2165 (40.3%)
Medicare only	3646 (39.3%)	1533 (45.5%)	233 (42.4%)	1880 (35.0%)
Medicaid only	533 (5.7%)	153 (4.5%)	22 (4.0%)	358 (6.7%)
Other	1808 (19.5%)	724 (21.5%)	115 (21.0%)	969 (18.0%)
Active on transplant waitlist	1048 (11.3%)	456 (13.5%)	37 (6.7%)	555 (10.3%)
Vascular access				
Fistula	5765 (62.1%)	2198 (65.2%)	349 (63.6%)	3218 (59.9%)
Graft	1974 (21.3%)	703 (20.9%)	127 (23.1%)	1144 (21.3%)
Catheter	1551 (16.7%)	468 (13.9%)	73 (13.3%)	1010 (18.8%)
Albumin (g/dL)	3.8 ± 0.4	3.9 ± 0.4	3.8 ± 0.4	3.8 ± 0.4
Hemoglobin (g/dL)	11.1 ± 1.2	11.2 ± 1.1	11.2 ± 1.0	11.1 ± 1.2
Kt/V	1.62 ± 0.28	1.63 ± 0.27	1.65 ± 0.28	1.61 ± 0.29
BMI (kg/m^2^)	28.4 ± 7.6	29.2 ± 7.6	28.0 ± 7.0	28.0 ± 7.5
Unexcused absences	1638 (17.6%)	476 (14.1%)	52 (9.5%)	1110 (20.7%)
Treatments shortened	4632 (49.9%)	1481 (44.0%)	204 (37.2%)	2947 (54.9%)
Hospitalizations	1303 (14.0%)	336 (10.0%)	59 (10.8%)	908 (16.9%)
ESRD vintage (months)	40.4 (19.5, 76.4)	37.6 (18.2, 72.1)	39.5 (18.6, 76.8)	42.5 (20.8, 78.5)
ESRD vintage > 12 months before current facility	2057 (22.1%)	711 (21.1%)	103 (18.8%)	1243 (23.1%)
Ability to ambulate	7105 (76.5%)	2858 (84.8%)	359 (65.4%)	3888 (72.4%)
Ability to transfer	7820 (84.2%)	3047 (90.4%)	415 (75.6%)	4358 (81.1%)
Falls	894 (9.6%)	312 (9.3%)	75 (13.7%)	507 (9.4%)
ADL score	5.7 ± 2.6	6.5 ± 2.1	4.4 ± 2.6	5.4 ± 2.8

Data shown as mean ± SD or median (25th, 75th percentiles) or n (%). *BMI* Body mass index, *ESRD* End-stage renal disease, *ADL* Activities of daily living

## Results

### Study population

**Fig. 2:**
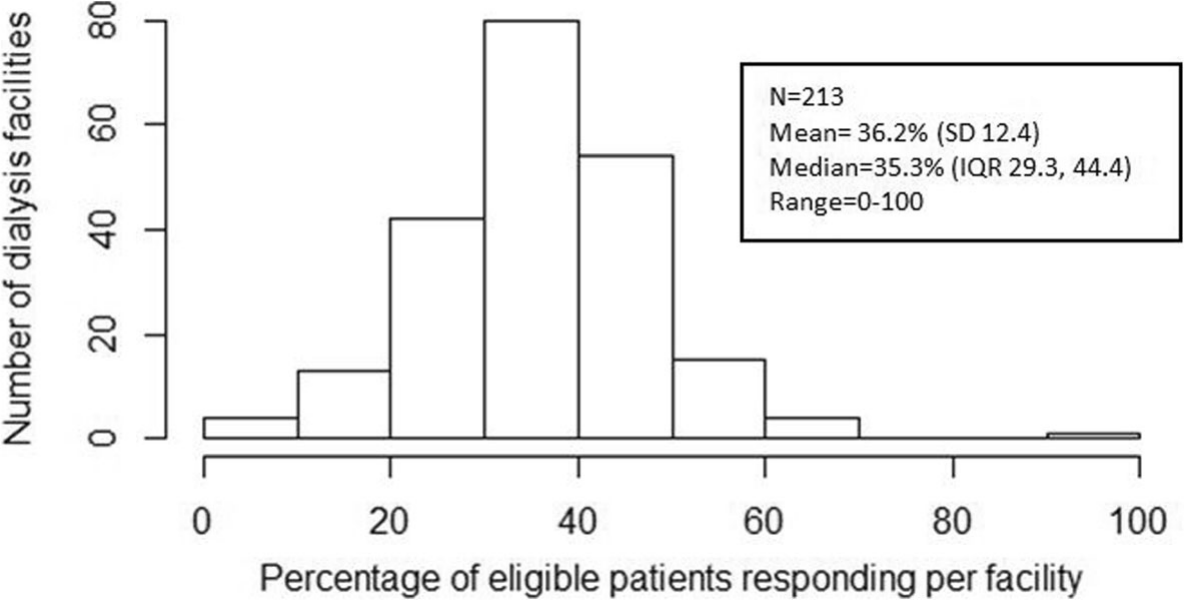
Distribution of Response Rates per Facility

There were 11,463 patients initially identified by DCI as meeting AHRQ-defined eligibility for the ICH CAHPS survey. Of these, 22 died during the survey administration period and 386 were deemed ineligible based on responses to eligibility screening questions in the survey meant to confirm ongoing in-center HD treatments at their HD facility for at least 3 months. The latter was probably a combination of incorrect initial identification, modality switch after identification, and inaccurate response from patients to the screening questions. Among 11,055 AHRQ eligible patients, 6541 (59%) did not return the survey or answer phone calls from the vendor. Of these non-responders, an additional 1169 (18%) patients were excluded in our primary analysis because of missing data on at least one covariate (Fig. 1). The response rate per facility ranged from 0 to 100% of eligible patients and narrowed to 15-61% for facilities with at least 30 survey eligible patients (Fig. 2). Of the 4514 (41%) patients who completed the survey, 643 (14%) responses could not be scored because of indicating proxy help or not completing at least 50% of the AHRQ key questions. Of all patients who provided any response, 596 (13%) were excluded in our primary analysis because of missing data on at least one covariate.

### Primary analyses

**Fig. 3:**
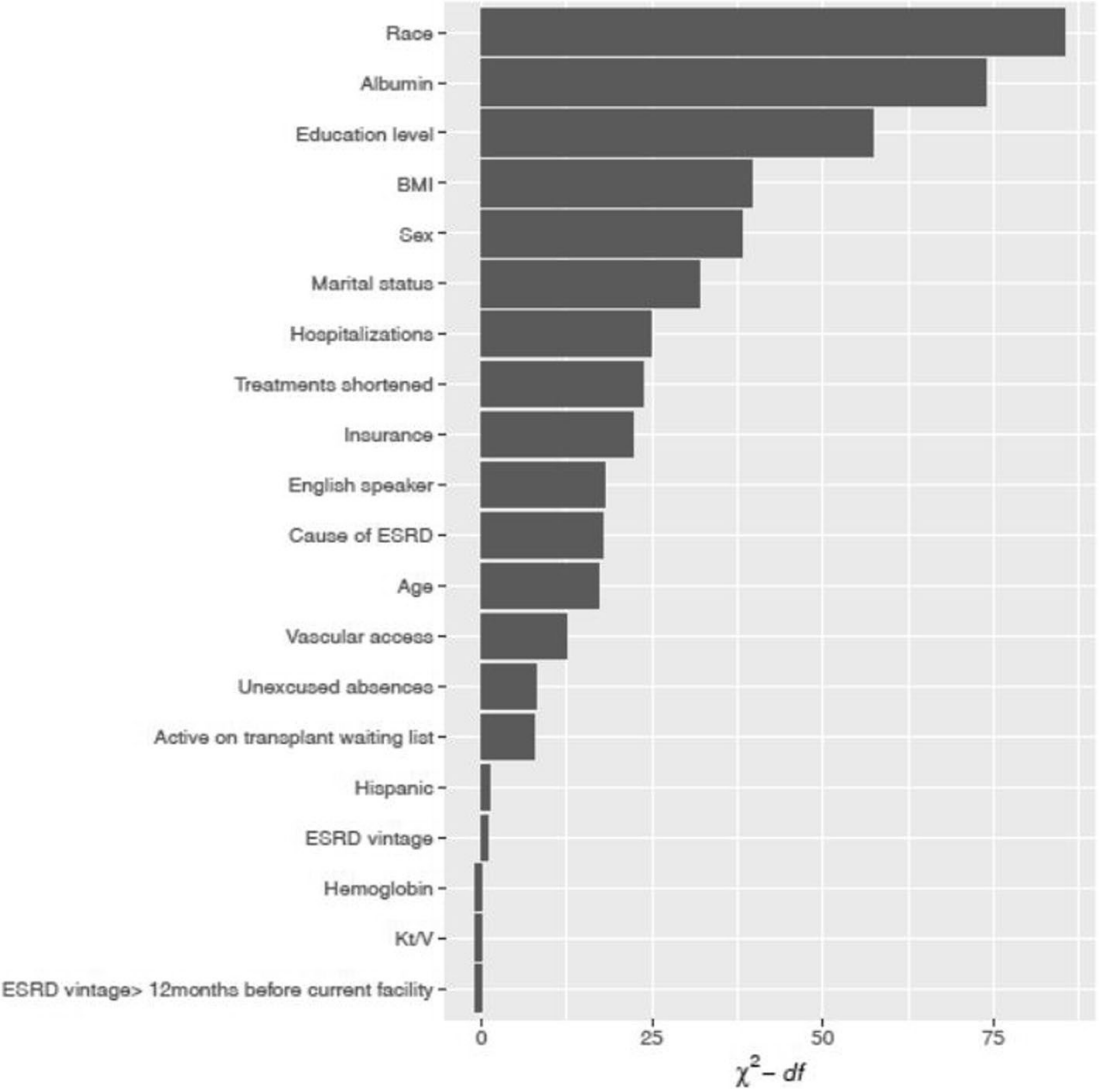
Ranking of variable contribution for determining non-response. Derived using data from model 2 (without functional covariates) shown in Table 2. ESRD: End stage renal disease; BMI: Body mass index. ESRD: End stage renal disease; ADL: Activities of daily living; BMI: Body mass index

Non-responders, based on AHRQ criteria, differed from responders and from those who had incomplete responses or proxy help in demographic, clinical, and functional characteristics (Table 1). In adjusted analyses, non-responders as compared to responders were more likely to be men, non-white, younger, single, dual Medicare/Medicaid eligible, less educated, and non-English speaking. Non-responders had longer ESRD vintage, were more likely to be inactive on the kidney transplant list, and had lower BMI and lower serum albumin. Non-responders had worse functional status, more hospitalizations, missed treatments, and shortened treatments (Table 2). Race, serum albumin concentration, and education level were the three most influential variables predicting non-response (Fig. 3).

**Table 2:** Multivariable logistic regression models predicting non-response

	Model 1	Model 2	Model 3
ICC	0.008	0.007	0.008
Age (per 5 years)	0.98 (0.96, 1.00)	**0.96 (0.94, 0.98)**	**0.90 (0.88, 0.92)**
Female Sex	**0.76 (0.69, 0.83)**	**0.73 (0.66, 0.80)**	**0.70 (0.63, 0.77)**
Race			
White	**0.64 (0.58, 0.71)**	**0.66 (0.59, 0.73)**	**0.60 (0.54, 0.67)**
Other	1.20 (0.97, 1.47)	1.20 (0.97, 1.49)	1.15 (0.92, 1.43)
Black	Ref	Ref	Ref
Hispanic ethnicity	1.17 (0.95, 1.45)	1.16 (0.93, 1.43)	1.20 (0.96, 1.49)
Insurance			
Medicare/Medicaid	**1.36 (1.19, 1.55)**	**1.27 (1.11, 1.45)**	1.07 (0.93, 1.23)
Medicare only	0.96 (0.85, 1.08)	0.96 (0.85, 1.08)	0.95 (0.84, 1.08)
Medicaid only	1.22 (0.97, 1.53)	1.10 (0.87, 1.38)	0.90 (0.71, 1.14)
Other	Ref	Ref	Ref
Marital status			
Married	**0.85 (0.74, 0.96)**	**0.87 (0.77, 1.00)**	**0.87 (0.76, 0.99)**
Divorced/separated	0.96 (0.84, 1.10)	0.94 (0.82, 1.08)	1.01 (0.87, 1.16)
Widowed	**1.32 (1.11, 1.56)**	**1.34 (1.12, 1.59)**	**1.35 (1.13, 1.61)**
Single	Ref	Ref	Ref
Education			
Grade school	**2.01 (1.69, 2.38)**	**1.97 (1.65, 2.34)**	**1.90 (1.59, 2.27)**
High school	**1.18 (1.06, 1.30)**	**1.14 (1.03, 1.27)**	**1.15 (1.04, 1.28)**
College or more	Ref	Ref	Ref
English speaker	**0.49 (0.34, 0.70)**	**0.45 (0.31, 0.65)**	**0.47 (0.32, 0.68)**
Hospitalization in last month		**1.43 (1.24, 1.65)**	**1.38 (1.19, 1.60)**
Active on transplant waitlist		**0.81 (0.70, 0.93)**	0.91 (0.79, 1.06)
BMI (per 2 kg/m^2^)		**0.96 (0.95, 0.97)**	**0.96 (0.95, 0.97)**
Cause ESRD			
Diabetes		**1.30 (1.16, 1.45)**	**1.15 (1.02, 1.29)**
Hypertension		1.09 (0.97, 1.23)	1.10 (0.97, 1.25)
Other		Ref	Ref
Vascular access			
Catheter		**1.29 (1.13, 1.47)**	1.06 (0.93, 1.22)
Graft		1.00 (0.89, 1.12)	0.96 (0.86, 1.09)
Fistula		Ref	Ref
Hemoglobin (per 0.5 g/dL)		0.99 (0.97, 1.01)	0.99 (0.97, 1.01)
Albumin (per 0.2 g/dL)		**0.89 (0.87, 0.92)**	**0.94 (0.92, 0.97)**
Kt/V (per 0.2)		1.00 (0.96, 1.04)	0.99 (0.95, 1.02)
ESRD vintage (per 12 months)		**1.02 (1.00, 1.04)**	**1.02 (1.00, 1.04)**
ESRD vintage > 12 months before current facility		0.96 (0.85, 1.09)	0.93 (0.82, 1.06)
Unexcused absences in last month		**1.22 (1.07, 1.38)**	**1.26 (1.11, 1.43)**
Treatments shortened in last month		**1.26 (1.15, 1.38)**	**1.26 (1.14, 1.38)**
Ability to ambulate			**0.83 (0.69, 1.00)**
Ability to transfer			1.15 (0.93, 1.41)
Falls in last month			0.93 (0.79, 1.09)
Nursing home resident			**1.77 (1.37, 2.29)**
ADL score (per 1 increase)			**0.83 (0.80, 0.85)**

Data shown as odds ratio (OR) (95% CI). Odds ratio above 1.00 is associated with non-response. Associations with *p* < 0.05 are in bold. Each model includes all of the covariates that have ORs listed. *BMI* Body mass index, *ESRD* End-stage renal disease, *ADL* Activities of daily living

### Secondary analyses

We included 549 survey responses with complete covariate data that either indicated receiving proxy help or did not respond to at least 50% of AHRQ pre-defined key questions (Additional file 1: Figure S1a). Overall, these 549 patients differed from AHRQ-defined responders in several demographic, clinical, and functional characteristics (Table 1). By including these surveys, we gained an average of 5% more responses for most demographic covariates (Additional file 1: Figure S2). Factors that predict non-response to the survey were similar to primary analyses when including this expanded response group (Additional file 1: Table S1).

### Sensitivity analyses

The majority of missing data was on functional covariates (Additional file 1: Table S2). Patients with missing covariate data were more often black and had shorter ESRD vintage (Additional file 1: Table S3). Models using multiple imputation for missing data had similar results to the primary and secondary analyses (Additional file 1: Tables S4 and S5).

## Discussion

In a large national in-center HD population, non-responders to the ICH CAHPS survey differed substantially from responders. Specifically, non-responders were more likely to be men, non-white, younger, single, dual Medicare/Medicaid eligible, less educated, non-English speaking, inactive on the transplant list, and had longer ESRD vintage, lower BMI and lower serum albumin, worse functional status, and more hospitalizations, missed treatments, and shortened treatments. These results demonstrate underrepresentation of important groups of in-center HD patients, broadly spanning individuals with fewer socioeconomic advantages and greater illness burden. It is possible that these results could introduce biases into facility-level ICH CAHPS survey results, particularly given low overall response rates, resulting in missed opportunities to assess and improve patient experience among the most vulnerable hemodialysis patients.

CAHPS surveys are widely used in US medical settings to evaluate patient experience, with other CAHPS surveys targeting hospitals, nursing homes and other settings. The ICH CAHPS is unique as it evaluates facilities with a relatively low number of patients per facility and with longstanding patient-facility relationships rather than discreet episodes. There is limited published literature on characteristics of non-responders to other CAHPS surveys. Most importantly, previous assessments use only limited patient-reported characteristics unlike our study where we use extensive characteristics gathered using reliable data sources rather than patient self-report. Even so, similar to our findings, analysis of Medicare Managed Care (MMC) CAHPS survey from 1997 and 1999 found significantly higher non-response rates in participants who were male and non-white [15]. Likewise, analysis of the Hospital CAHPS (HCAHPS) pilot survey data from 2002 to 2003 also found male sex, younger age, and non-white race to be significantly associated with non-response [16]. Finally, in a large sample of Medicare CAHPS participants from 2007 there were significantly higher non-response rates in participants who were men, non-white, and dual eligible for Medicare and Medicaid [17].

These results have important implications for assessment of dialysis patient experience. The DCI patient population is similar to the broader US dialysis population with the exception that there is a slightly higher proportion of black patients receiving dialysis care at DCI [18]. To our knowledge, this is the only non-CMS dataset linking individual patient-level clinical data to ICH CAHPS responses, and this is the first study assessing the differences in characteristics, including laboratory variables and treatment characteristics, between responders and non-responders. Previous published work on the ICH CAHPS survey is restricted to reports on the development and testing of this survey, where there was a response rate of 46% and there was no published evaluation of non-responders [8, 19]. Within one of these study cohorts, response rates were noted to be higher among those assigned to mail followed by telephone versus telephone only survey administration [20]. Although supervision of ICH CAHPS administration was transferred from AHRQ to CMS in 2014, the current survey remains similar to the one administered in 2012, with the major exception that limited assistance is now allowed, consistent with the ‘expanded usable’ criteria used in secondary analyses in this manuscript.

Payers increasingly are moving towards value based purchasing models, with performance metrics critical to quantify value. Before the addition of ICH CAHPS as a performance metric, the ESRD QIP was composed of only clinical and laboratory measures, most of which were not specifically patient-centered outcomes [21]. Patient experience measures have been widely implemented in other areas of healthcare, and use of the ICH CAHPS survey represents an important milestone for in-center HD; however, attempts to address patient-centered care using a patient-reported outcome measures with low response rates may have limitations. Paradoxically, we found that non-responders tended to be patients who are disproportionately represented in the US ESRD population as compared to the general population (specifically younger, black, male, and diabetics) [18]. These differences in characteristics associated with non-response raise the possibility of non-response bias; however further research is needed in evaluating whether or not these characteristics are also associated with experience scores and will thereby affect facility performance ratings and performance-based payments as well as misrepresent key areas needed for intervention to improve patient experience [22].

The specific reasons for non-response remain unknown. Neither the former AHRQ nor the current CMS administration process collects reasons for non-response unless it is due to incorrect contact information. Comorbid conditions common among dialysis patients include physical, cognitive, and visual impairments that may limit the ability of HD patients to respond to a survey themselves. Accordingly, and particularly in view of the survey’s length (currently 62 questions), the initial decision by AHRQ to not allow any assistance may have had important implications. Using a less restrictive method of classifying survey completion, more consistent with current CMS guidance, we were able to include approximately 5% more responses across most demographic characteristics; notably, inclusion of these surveys did not change the predictors of non-response.

Our results may have substantial implications for dialysis facilities if characteristics associated with non-response are also associated with experience scores. Starting in calendar year 2016 (and reflected in 2018 payments to facilities), survey results are a clinical performance measure within the ESRD QIP and experience scores can impact facility payments from CMS [9].

An important strength of this study is that it documents new information about the real-world administration of the ICH CAHPS survey. Additionally, this study provides information that can no longer be gathered since survey vendors are now barred from providing patient-level data to dialysis facilities. Other strengths include having a large number of survey responses from a national dialysis provider linked to extensive facility gathered patient-level demographic, clinical, and functional data. Limitations include not knowing the precise date of survey completion during the survey administration period, which required the use of proximate covariate data. As with most surveys, we do not have information on reasons for non-response.

ICH CAHPS survey response rates remain low overall (only 33% despite allowing limited assistance with survey completion [10]). Future studies should provide ongoing evaluation into the presence of and reasons for non-response to this survey to inform strategies for engaging populations that have a greater likelihood of non-response, specifically patients with greater illness burden and fewer socioeconomic advantages, in order to improve the generalizability and utility of surveys of patient experience.

## Conclusion

There are significant differences between ICH CAHPS survey non-responders and responders that potentially affect facility-level assessment of and efforts to better patient experience. Further work should evaluate causes of non-response and interventions to increase response rates in an attempt to optimize assessment and improve patient experience.

## Additional file

Additional file 1: **Box 1.** Key questions from the 2012 ICH CAHPS survey. **Figure S1a.** Flow diagram for secondary analysis. **Figure S1b.** Flow diagram for first sensitivity analysis. **Figure S1c.** Flow diagram for second sensitivity analysis. **Figure S2.** Responses gained using the expanded usable criteria. **Table S1.** Multivariable logistic regression models predicting non-response using “expanded usable criteria”. **Table S2.** Proportion of missing data for each covariate. **Table S3.** Baseline characteristics of participants with complete vs missing data. **Table S4.** Multivariable logistic regression models predicting non-response using multiple imputation using AHRQ method . **Table S5.** Multivariable logistic regression models predicting non-response using imputation using “expanded usable criteria”. **Figure S3a.** Ranking of variable contribution for determining non-response using AHRQ method and model 3. **Figure S3b.** Ranking of variable contribution for determining non-response using “expanded usable” criteria and model 2. **Figure S3c.** Ranking of variable contribution for determining non-response using “expanded usable” criteria and model 3. (PDF 743 kb)
