# Supplementary material for: Evaluation of non-response to the In-Center Hemodialysis Consumer Assessment of Healthcare Providers and Systems (ICH CAHPS) survey
Source: BMC Health Serv Res. 2018 Oct 19;18:790. doi: 10.1186/s12913-018-3618-4 (PMC6194668; doi:10.1186/s12913-018-3618-4)
Supplement: Supplementary file 1 — Box 1. Key questions from the 2012 ICH CAHPS survey. Figure S1a. Flow diagram for secondary analysis. Figure S1b. Flow diagram for first sensitivity analysis. Figure S1c. Flow diagram for second sensitivity analysis. Figure S2. Responses gained using the expanded usable criteria. Table S1. Multivariable logistic regression models predicting non-response using “expanded usable criteria”. Table S2. Proportion of missing data for each covariate. Table S3. Baseline characteristics of participants with complete vs missing data. Table S4. Multivariable logistic regression models predicting non-response using multiple imputation using AHRQ method . Table S5. Multivariable logistic regression models predicting non-response using imputation using “expanded usable criteria”. Figure S3a. Ranking of variable contribution for determining non-response using AHRQ method and model 3. Figure S3b. Ranking of variable contribution for determining non-response using “expanded usable” criteria and model 2. Figure S3c. Ranking of variable contribution for determining non-response using “expanded usable” criteria and model 3. (PDF 743 kb) [file 12913_2018_3618_MOESM1_ESM.pdf]

Supplementary material

Supplementary Box 1: Key questions from the 2012 ICH CAHPS survey

Question number	Question wording
1	Where do you get your dialysis treatments?
2	How long have you been getting dialysis at this dialysis center?
8	Using any number from 0 to 10, where 0 is the worst kidney doctors possible and 10 is the best kidney doctors possible, what number would you use to rate the kidney doctors you have now?
20	In the last 3 months, which one did they use most often to connect you to the dialysis machine?
23	In the last 3 months, did any problems occur during your dialysis?
32	Using any number from 0 to 10, where 0 is the worst dialysis center staff possible and 10 is the best dialysis center staff possible, what number would you use to rate your dialysis center staff?
35	Using any number from 0 to 10, where 0 is the worst dialysis center possible and 10 is the best dialysis center possible, what number would you use to rate your dialysis center?
37	Are you eligible for a kidney transplant?
41	In the last 12 months, were you ever unhappy with the care you received at the dialysis center or from your kidney doctors?
45	In general, how would you rate your overall health?
46	In general, how would you rate your overall mental or emotional health?
47	Are you being treated for high blood pressure?
48	Are you being treated for diabetes or high blood sugar?
49	Are you being treated for heart disease or heart problems?
50	What is your age?
51	Are you male or female?
52	What is the highest grade or level of school that you have completed?
53	Are you of Hispanic or Latino origin or descent?
54	What is your race? Please mark one or more.
55	What language do you mainly speak at home?
56	Did someone help you complete this survey?

*Responses to at least 12 of these questions were required for the survey to be deemed usable

Supplementary Figure 1a: Flow diagram for secondary analysis

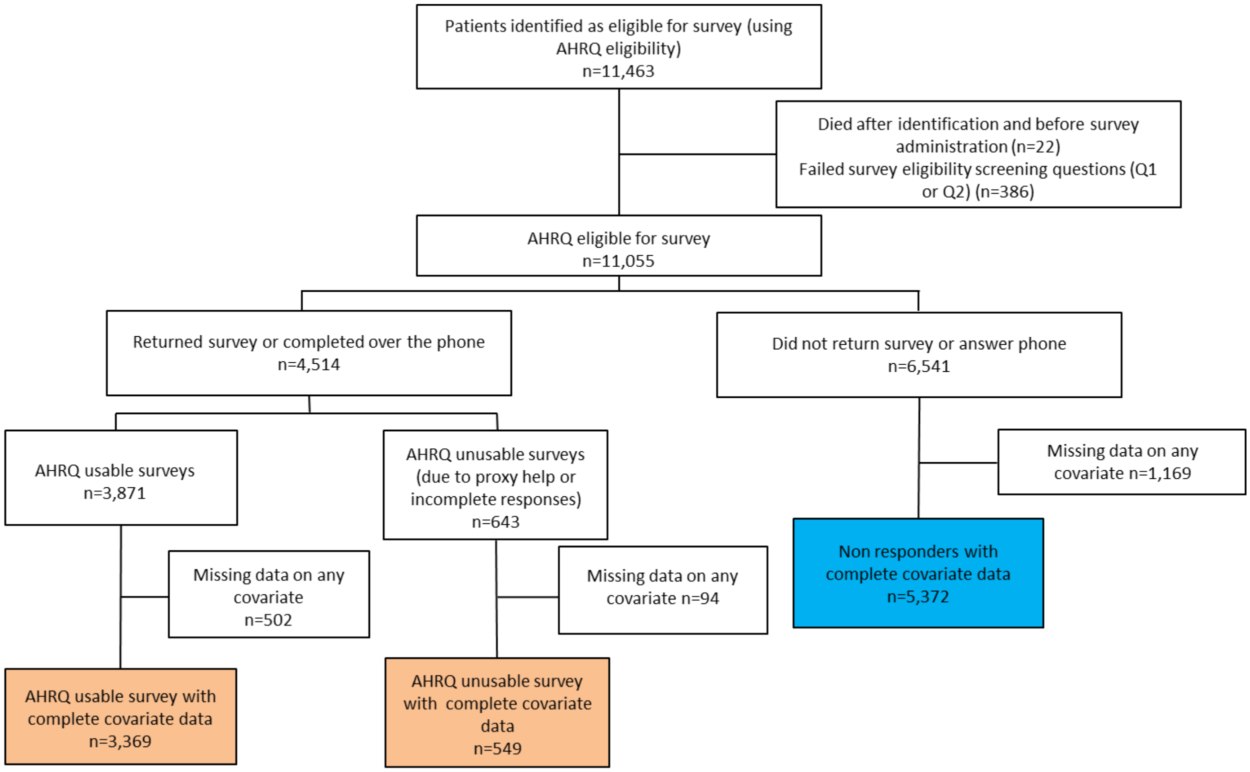

*Patients in the orange boxes are combined (“Expanded usable”) and compared to patients in the blue box above

Supplementary Figure 1b: Flow diagram for first sensitivity analysis

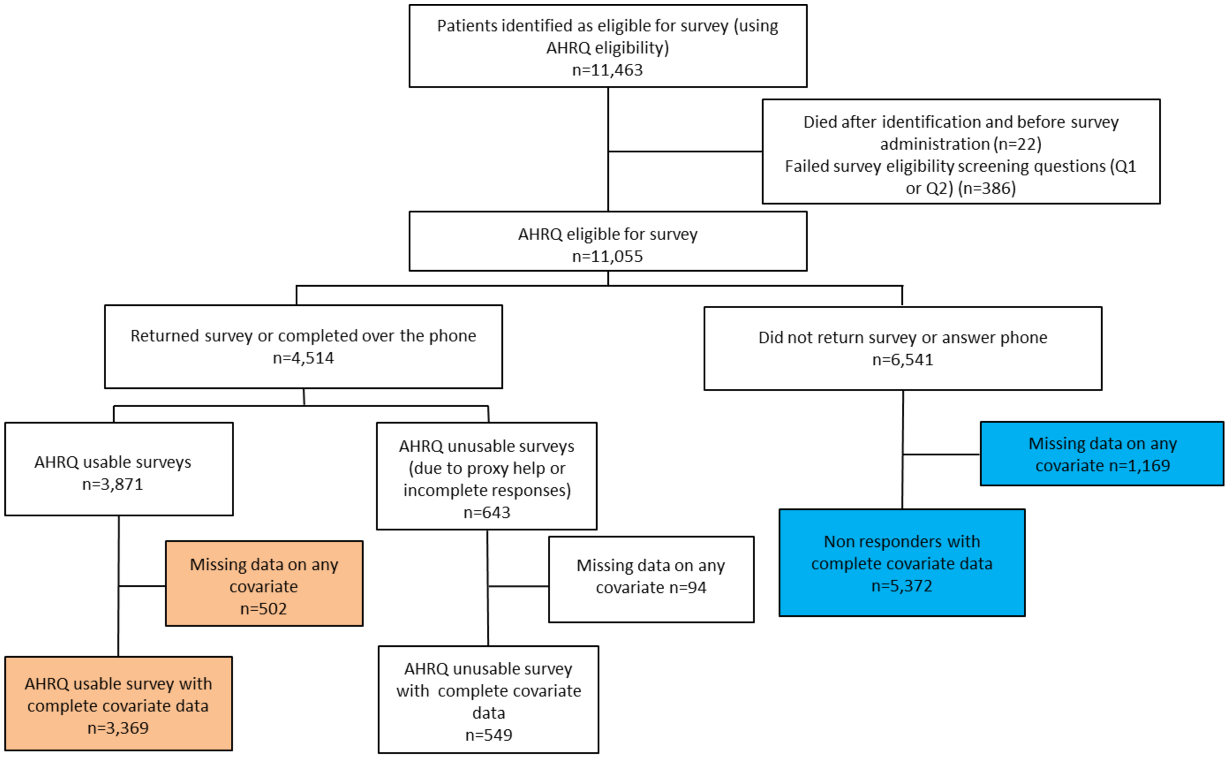

*After multiple imputation for missing non-functional covariate data, patients from the orange boxes are compared to patients from the blue boxes above

Supplementary Figure 1c: Flow diagram for second sensitivity analysis

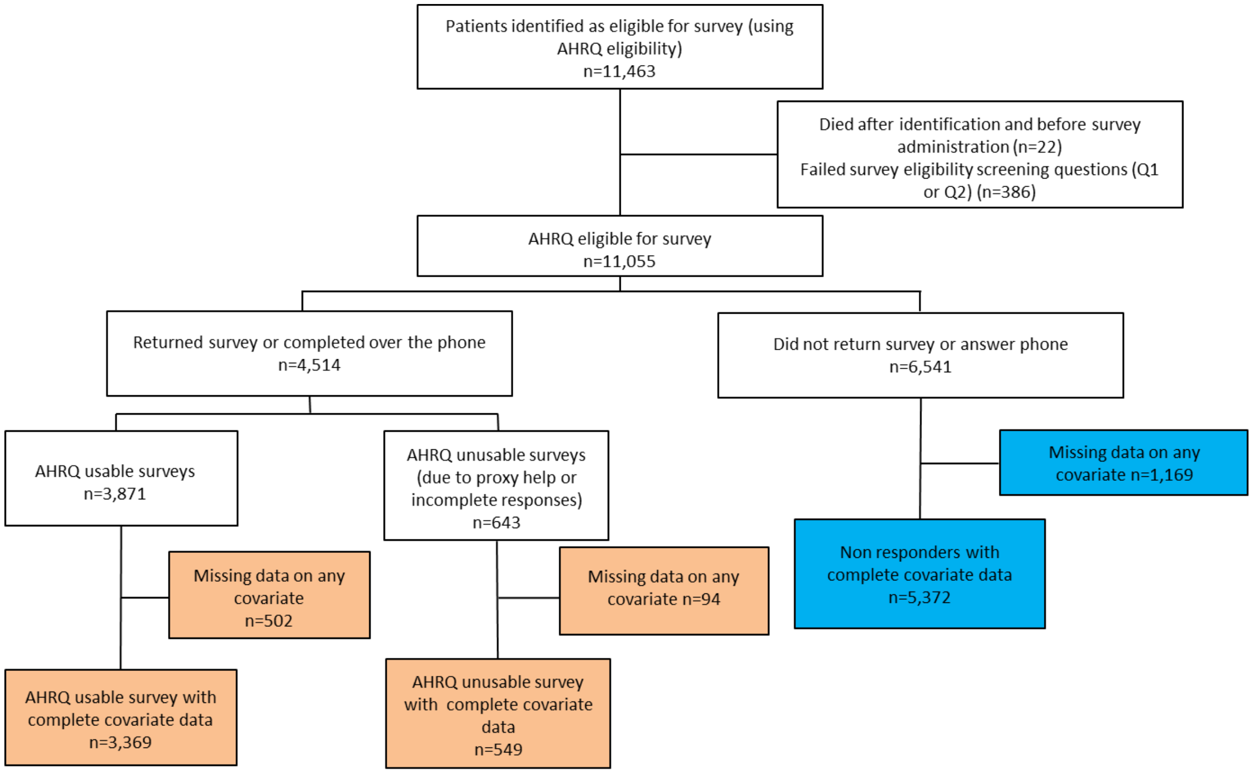

*After multiple imputation for missing non-functional covariate data, patients from the orange boxes are compared to patients from the blue boxes above

Supplementary Figure 2: Responses gained using the expanded usable criteria

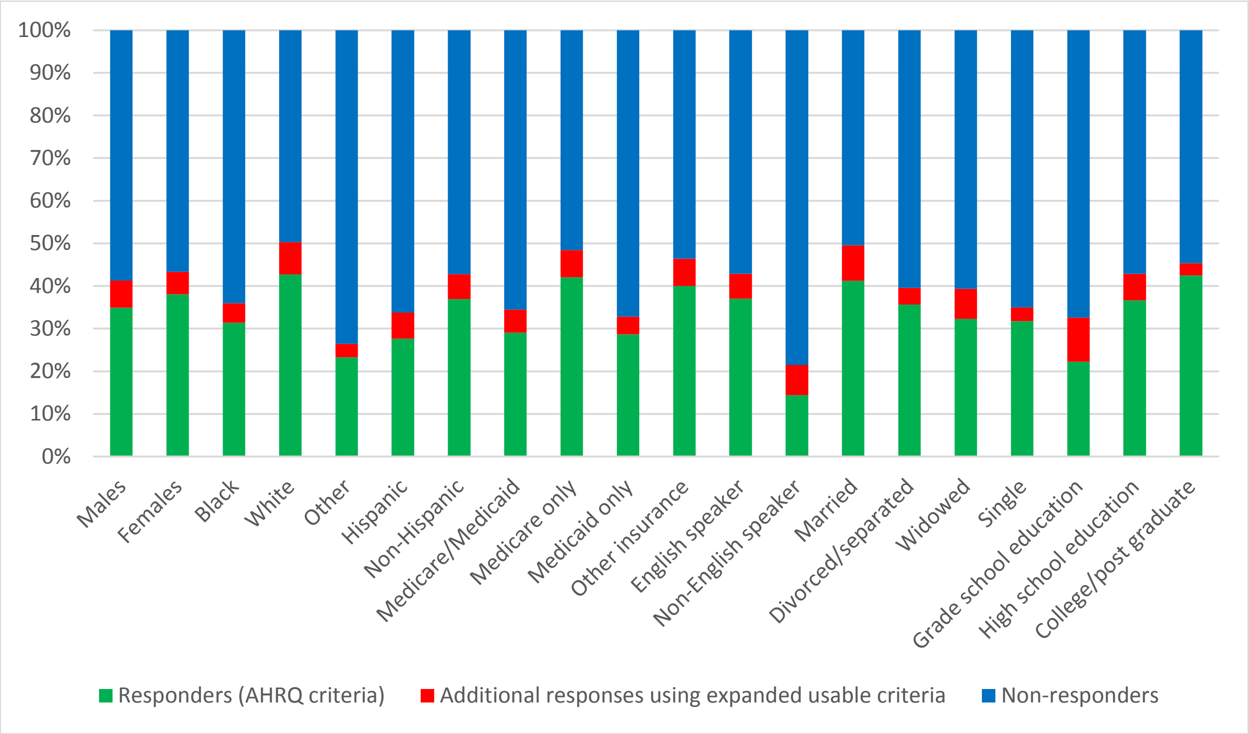

Supplementary Table 1: Multivariable logistic regression models predicting non-response using "expanded usable" criteria

	Model 1	Model 2	Model 3
ICC	0.012	0.013	0.014
Age, per 5 years	**0.95 (0.93, 0.97)**	**0.93 (0.91, 0.95)**	**0.89 (0.87, 0.91)**
Female sex	**0.81 (0.74, 0.88)**	**0.79 (0.72, 0.88)**	**0.77 (0.70, 0.86)**
Race, white vs. black	**0.61 (0.55, 0.67)**	**0.62 (0.55, 0.68)**	**0.58 (0.52, 0.65)**
Race, other vs. black	**1.33 (1.08, 1.63)**	**1.37 (1.11, 1.69)**	**1.34 (1.08, 1.66)**
Ethnicity, Hispanic vs non-Hispanic	1.17 (0.95, 1.43)	1.17 (0.95, 1.45)	1.21 (0.98, 1.49)
Insurance, Medicare/Medicaid vs. other	**1.27 (1.12, 1.45)**	**1.20 (1.05, 1.37)**	1.04 (0.91, 1.20)
Insurance, Medicare only vs. other	0.97 (0.87, 1.10)	0.99 (0.88, 1.12)	0.99 (0.87, 1.12)
Insurance, Medicaid only vs. other	1.16 (0.93, 1.45)	1.05 (0.84, 1.31)	0.89 (0.71, 1.13)
Marital status, married vs. single	**0.75 (0.66, 0.85)**	**0.77 (0.68, 0.88)**	**0.78 (0.69, 0.89)**
Marital status, divorced/separated vs. single	1.00 (0.88, 1.14)	0.98 (0.86, 1.12)	1.03 (0.90, 1.19)
Marital status, widowed vs. single	**1.31 (1.11, 1.54)**	**1.33 (1.12, 1.57)**	**1.34 (1.13, 1.59)**
Education level, grade school vs. college or more	**1.36 (1.16, 1.60)**	**1.33 (1.13, 1.57)**	**1.29 (1.10, 1.53)**
Education level, high school vs. college or more	1.00 (0.91, 1.11)	0.98 (0.88, 1.09)	0.99 (0.89, 1.10)
English speaker	**0.52 (0.38, 0.72)**	**0.48 (0.35, 0.66)**	**0.49 (0.35, 0.68)**
Hospitalizations		**1.49 (1.30, 1.71)**	**1.45 (1.26, 1.67)**
Active on transplant waitlist		**0.82 (0.71, 0.95)**	0.90 (0.77, 1.04)
BMI, per 2 kg/m^2		**0.96 (0.95, 0.97)**	**0.96 (0.95, 0.97)**
Cause ESRD, diabetes vs. other		**1.17 (1.05, 1.31)**	1.07 (0.95, 1.20)
Cause ESRD, hypertension vs. other		1.07 (0.95, 1.21)	1.07 (0.95, 1.21)
Vascular access, catheter vs. fistula		1.34 (1.18, 1.53)	**1.16 (1.02, 1.32)**
Vascular access, graft vs. fistula		0.98 (0.88, 1.10)	0.96 (0.85, 1.08)
Hemoglobin, per 0.5 g/dL		0.99 (0.97, 1.01)	0.99 (0.97, 1.01)
Albumin, per 0.2 g/dL		**0.92 (0.89, 0.94)**	**0.95 (0.93, 0.98)**
Kt/V, per 0.2		0.98 (0.95, 1.02)	0.97 (0.94, 1.01)
ESRD vintage, per 12 months		1.01 (1.00, 1.03)	1.01 (1.00, 1.03)
ESRD vintage > 12 months before current facility		0.97 (0.86, 1.10)	0.95 (0.84, 1.07)
Unexcused absences		**1.30 (1.15, 1.47)**	**1.35 (1.19, 1.53)**
Treatments shortened		**1.33 (1.21, 1.45)**	**1.32 (1.20, 1.45)**
Ability to ambulate			**0.84 (0.71, 1.00)**
Ability to transfer			1.16 (0.95, 1.41)
Falls			0.88 (0.76, 1.03)
Nursing home resident			**2.11 (1.68, 2.64)**
ADL score, per 1 increase			**0.88 (0.86, 0.91)**

Data shown as odds ratio (OR) (95% CI). OR above 1.00 is associated with non-response. Associations with p<0.05 are in bold. BMI: Body mass index; ESRD: End stage renal disease; ADL: Activities of daily living

Supplementary Table 2: Proportion of missing data for each covariate

	Eligible (N=11055)
Race	149 (1.4%)
Ethnicity	194 (1.8%)
ESRD cause	14 (0.1%)
Insurance	287 (2.6%)
Vascular access	16 (0.1%)
English speaker	597 (5.4%)
Nursing Home Residence	631 (5.7%)
Ability to ambulate	597 (5.4%)
Ability to transfer	597 (5.4%)
Falls	597 (5.4%)
Marital status	478 (4.3%)
Education Level	478 (4.3%)
Transplant waitlist status	529 (4.8%)
Albumin	23 (0.2%)
Hemoglobin	19 (0.2%)
Kt/V	74 (0.7%)
ADL score	597 (5.4%)
BMI	148 (1.3%)

Data shown as n (%). BMI: Body mass index; ESRD: End stage renal disease; ADL: Activities of daily living

Supplementary Table 3: Baseline characteristics of participants with complete vs missing data

	Eligible (N=11055)	Any missing data (n=1765, 16%)	Complete data (n=9290, 84%)	***p***
Age	61.0 ± 14.8	60.4 ± 14.7	61.1 ± 14.8	0.04
Female sex	4893 (44.3%)	825 (46.7%)	4068 (43.8%)	0.02
Race				
Black	5054 (46.3%)	928 (57.4%)	4126 (44.4%)	<0.001
White	5071 (46.5%)	585 (36.2%)	4486 (48.3%)	
Other	781 (7.2%)	103 (6.4%)	678 (7.3%)	
Hispanic Ethnicity	748 (6.9%)	111 (7.1%)	637 (6.9%)	0.76
Cause of ESRD				
Diabetes	4719 (42.7%)	704 (40.2%)	4015 (43.2%)	<0.001
Hypertension	3223 (29.2%)	593 (33.9%)	2630 (28.3%)	
Other	3099 (28.1%)	454 (25.9%)	2645 (28.5%)	
Marital status				
Married	3988 (37.7%)	433 (33.6%)	3555 (38.3%)	0.002
Divorced/Separated	2209 (20.9%)	262 (20.4%)	1947 (21.0%)	
Widowed	1693 (16.0%)	217 (16.9%)	1476 (15.9%)	
Single	2687 (25.4%)	375 (29.1%)	2312 (24.9%)	
Education Level				
Grade School	1397 (13.2%)	176 (13.7%)	1221 (13.1%)	0.86
High School	6463 (61.1%)	784 (60.9%)	5679 (61.1%)	
College/Post Graduate	2717 (25.7%)	327 (25.4%)	2390 (25.7%)	
English speaker	10130 (96.9%)	1138 (97.4%)	8992 (96.8%)	0.24
Nursing home resident	769 (7.4%)	87 (7.7%)	682 (7.3%)	0.69
Insurance				
Medicare/Medicaid	3812 (35.4%)	509 (34.4%)	3303 (35.6%)	0.03
Medicare only	4272 (39.7%)	626 (42.4%)	3646 (39.3%)	
Medicaid only	627 (5.8%)	94 (6.4%)	533 (5.7%)	
Other	2057 (19.1%)	249 (16.9%)	1808 (19.5%)	
Active on transplant waitlist	1199 (11.4%)	151 (12.2%)	1048 (11.3%)	0.33
Vascular access				
Fistula	6773 (61.4%)	1008 (57.6%)	5765 (62.1%)	<0.001
Catheter	1922 (17.4%)	371 (21.2%)	1551 (16.7%)	
Graft	2344 (21.2%)	370 (21.2%)	1974 (21.3%)	
Albumin (g/dL)	3.8 ± 0.4	3.8 ± 0.5	3.8 ± 0.4	<0.001
Hemoglobin (g/dL)	11.1 ± 1.2	11.1 ± 1.3	11.1 ± 1.2	0.03
Kt/V	1.61 ± 0.28	1.57 ± 0.28	1.62 ± 0.28	<0.001
BMI (kg/m^2)	28.4 ± 7.6	28.5 ± 7.6	28.4 ± 7.6	0.65
Unexcused absences	1964 (17.8%)	326 (18.5%)	1638 (17.6%)	0.40
Treatments shortened	5485 (49.6%)	853 (48.3%)	4632 (49.9%)	0.24
Hospitalizations	1541 (13.9%)	238 (13.5%)	1303 (14.0%)	0.55
ESRD vintage (months)	39.4 (18.8, 75.3)	34.3 (14.9, 69.4)	40.4 (19.5, 76.4)	<0.001
ESRD vintage > 12 months before current facility	2413 (21.8%)	356 (20.2%)	2057 (22.1%)	0.07

Ability to ambulate	7970 (76.2%)	865 (74.1%)	7105 (76.5%)	0.07
Ability to transfer	8769 (83.9%)	949 (81.3%)	7820 (84.2%)	0.01
Falls	1017 (9.7%)	123 (10.5%)	894 (9.6%)	0.32
ADL score	5.7 ± 2.6	5.7 ± 2.6	5.7 ± 2.6	0.96

Data shown as mean ± SD or median (25th, 75th percentiles) or n (%). p-value using Chi-squared statistic for categorical variables t-test for all continuous variables except ESRD vintage which is based on the non-parametric Wilcoxon test. BMI: Body mass index; ESRD: End stage renal disease; ADL: Activities of daily living

Supplementary table 4: Multivariable logistic regression models predicting non-response using multiple imputation using AHRQ method

	Model 1	Model 2
Age, per 5 years	0.99 (0.97, 1.00)	**0.96 (0.94, 0.98)**
Female sex	**0.77 (0.71, 0.84)**	**0.72 (0.65, 0.79)**
Race, white vs. black	**0.63 (0.58, 0.70)**	**0.64 (0.58, 0.71)**
Race, other vs. black	1.16 (0.96, 1.42)	1.18 (0.97, 1.44)
Ethnicity, Hispanic vs non-Hispanic	1.17 (0.95, 1.44)	1.18 (0.95, 1.46)
Insurance, Medicare/Medicaid vs. other	**1.37 (1.21, 1.55)**	**1.28 (1.12, 1.45)**
Insurance, Medicare only vs. other	0.96 (0.86, 1.07)	0.96 (0.86, 1.08)
Insurance, Medicaid only vs. other	**1.32 (1.07, 1.63)**	1.20 (0.96, 1.49)
Marital status, married vs. single	**0.83 (0.73, 0.93)**	**0.86 (0.76, 0.97)**
Marital status, divorced/separated vs. single	0.95 (0.84, 1.08)	0.94 (0.82, 1.07)
Marital status, widowed vs. single	**1.24 (1.05, 1.45)**	**1.25 (1.06, 1.47)**
Education level, grade school vs. college or more	**2.08 (1.76, 2.46)**	**2.04 (1.72, 2.42)**
Education level, high school vs. college or more	**1.22 (1.11, 1.35)**	**1.19 (1.08, 1.32)**
English speaker	**0.53 (0.40, 0.70)**	**0.51 (0.38, 0.67)**
Hospitalizations		**1.46 (1.28, 1.67)**
Active on transplant waitlist		**0.82 (0.72, 0.94)**
BMI, per 2 kg/m^2		**0.96 (0.95, 0.97)**
Cause ESRD, diabetes vs. other		**1.32 (1.19, 1.47)**
Cause ESRD, hypertension vs. other		**1.13 (1.01, 1.27)**
Vascular access, catheter vs. fistula		**1.33 (1.18, 1.50)**
Vascular access, graft vs. fistula		1.02 (0.92, 1.14)
Hemoglobin, per 0.5 g/dL		0.99 (0.97, 1.01)
Albumin, per 0.2 g/dL		**0.89 (0.87, 0.91)**
Kt/V, per 0.2		1.02 (0.97, 1.08)
ESRD vintage, per 12 months		**1.02 (1.00, 1.04)**
ESRD vintage > 12 months before current facility		0.96 (0.86, 1.08)
Unexcused absences		**1.26 (1.12, 1.41)**
Treatments shortened		**1.24 (1.13, 1.35)**

Data shown as odds ratio (OR) (95% CI). OR above 1.00 is associated with non-response. Associations with p<0.05 are in bold. Only 2 models are show since we did not impute functional covariate data. BMI: Body mass index; ESRD: End stage renal disease; ADL: Activities of daily living

Supplementary table 5: Multivariable logistic regression models predicting non-response using multiple imputation using “expanded usable criteria”

	Model 1	Model 2
Age, per 5 years	**0.95 (0.94, 0.97)**	**0.93 (0.91, 0.95)**
Female sex	**0.82 (0.76, 0.89)**	**0.78 (0.71, 0.86)**
Race, white vs. black	**0.61 (0.56, 0.67)**	**0.61 (0.56, 0.68)**
Race, other vs. black	**1.26 (1.04, 1.53)**	**1.30 (1.06, 1.59)**
Ethnicity, Hispanic vs non-Hispanic	1.18 (0.98, 1.42)	1.20 (1.00, 1.45)
Insurance, Medicare/Medicaid vs. other	**1.29 (1.15, 1.46)**	**1.23 (1.08, 1.39)**
Insurance, Medicare only vs. other	0.96 (0.86, 1.08)	0.98 (0.87, 1.10)
Insurance, Medicaid only vs. other	**1.26 (1.03, 1.55)**	1.15 (0.93, 1.42)
Marital status, married vs. single	**0.76 (0.67, 0.85)**	**0.78 (0.69, 0.88)**
Marital status, divorced/separated vs. single	1.00 (0.88, 1.14)	0.99 (0.87, 1.13)
Marital status, widowed vs. single	**1.22 (1.05, 1.43)**	**1.24 (1.06, 1.45)**
Education level, grade school vs. college or more	**1.40 (1.21, 1.63)**	**1.37 (1.17, 1.60)**
Education level, high school vs. college or more	1.04 (0.95, 1.14)	1.01 (0.92, 1.12)
English speaker	**0.51 (0.40, 0.65)**	**0.49 (0.38, 0.62)**
Hospitalizations		**1.54 (1.35, 1.75)**
Active on transplant waitlist		**0.82 (0.71, 0.94)**
BMI, per 2 kg/m^2		**0.96 (0.95, 0.97)**
Cause ESRD, diabetes vs. other		**1.19 (1.07, 1.32)**
Cause ESRD, hypertension vs. other		1.11 (0.99, 1.24)
Vascular access, catheter vs. fistula		**1.41 (1.25, 1.59)**
Vascular access, graft vs. fistula		0.98 (0.88, 1.09)
Hemoglobin, per 0.5 g/dL		0.98 (0.95, 1.01)
Albumin, per 0.2 g/dL		**0.92 (0.88, 0.96)**
Kt/V, per 0.2		1.02 (0.96, 1.08)
ESRD vintage, per 12 months		1.01 (0.99, 1.03)
ESRD vintage > 12 months before current facility		0.98 (0.88, 1.10)
Unexcused absences		**1.34 (1.19, 1.50)**
Treatments shortened		**1.27 (1.17, 1.38)**

Data shown as odds ratio (OR) (95% CI). OR above 1.00 is associated with non-response. Associations with $p<0.05$ are in bold. BMI: Body mass index; ESRD: End stage renal disease; ADL: Activities of daily living

Supplementary Figure 3a: Ranking of variable contribution for determining non-response using AHRQ method and full model (Model 3)

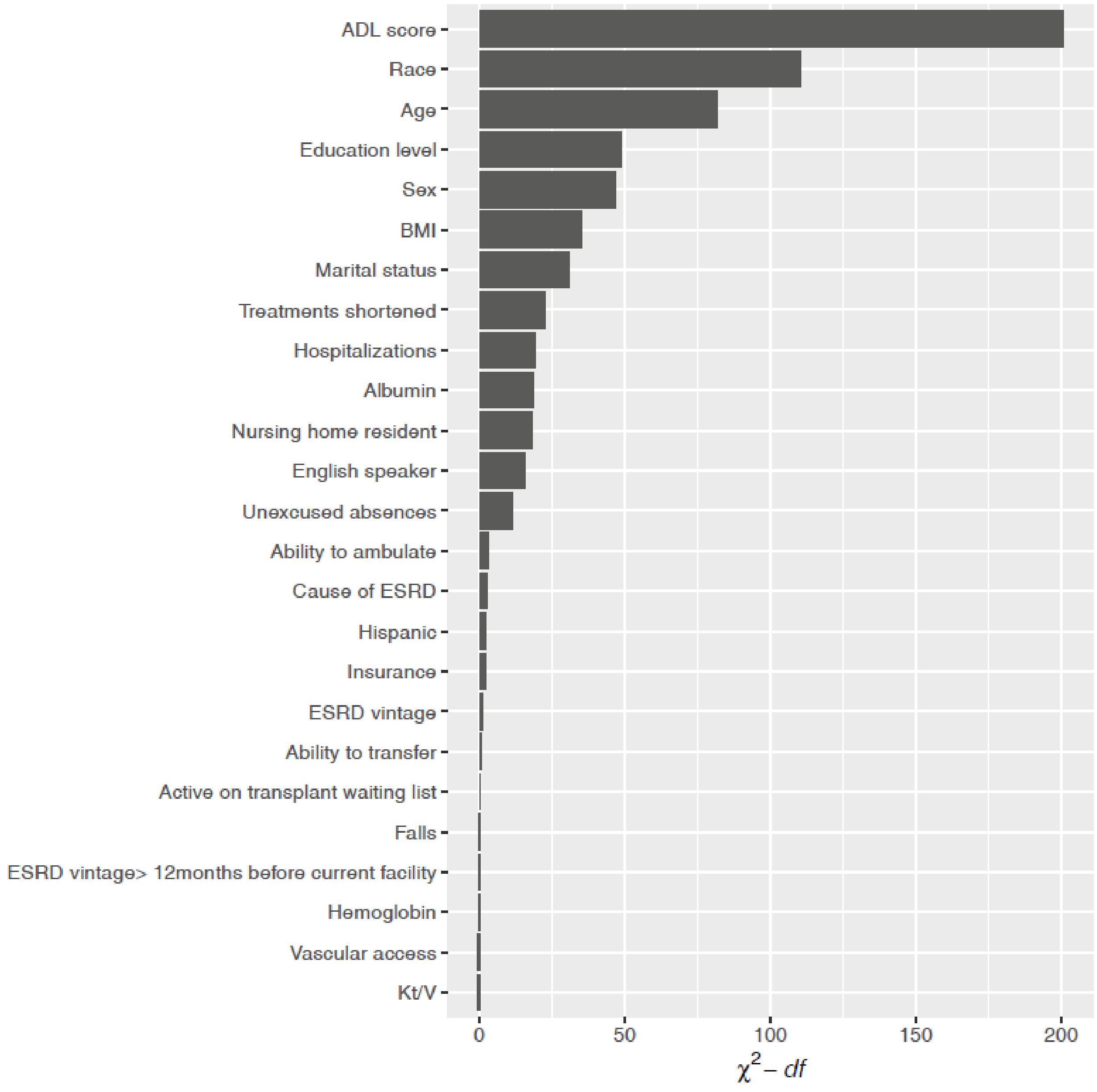

ESRD: End stage renal disease; ADL: Activities of daily living; BMI: Body mass index

Supplementary Figure 3b: Ranking of variable contribution for determining non-response using "expanded usable" criteria and model 2 (without functional covariates)

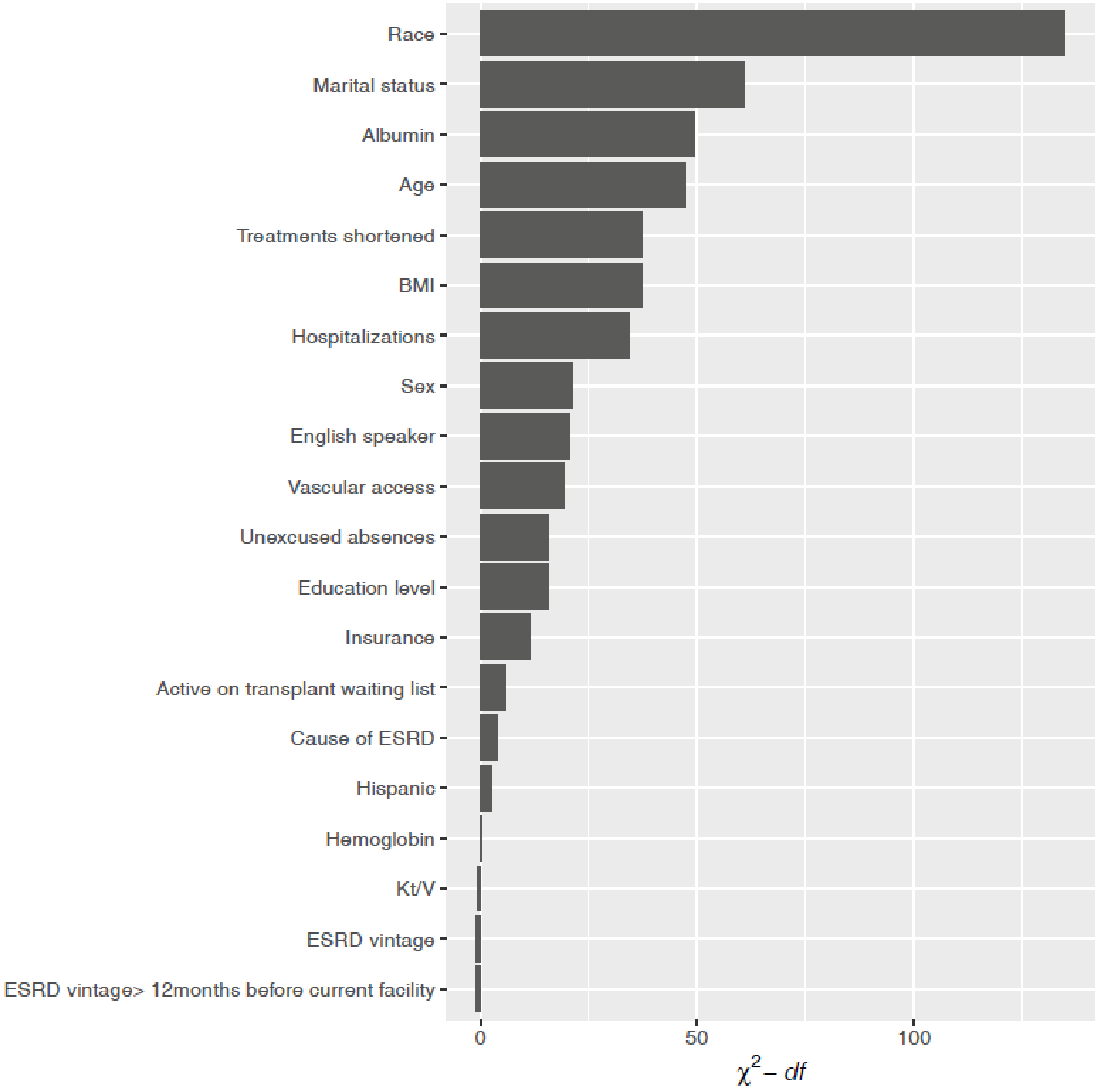

ESRD: End stage renal disease; BMI: Body mass index

Supplementary Figure 3c: Ranking of variable contribution for determining non-response using "expanded usable" criteria and full model (Model 3)

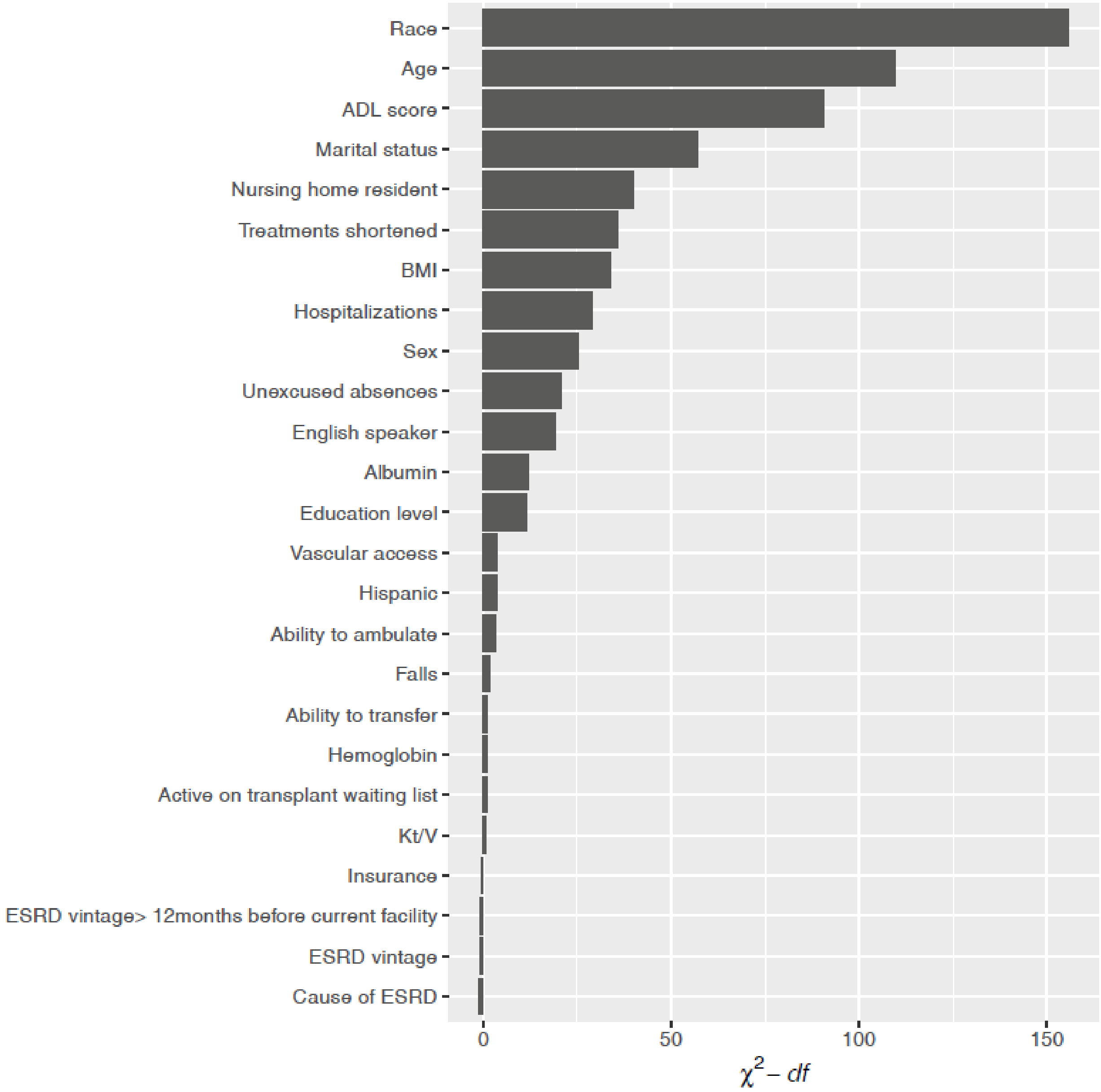

ESRD: End stage renal disease; ADL: Activities of daily living; BMI: Body mass index
